# Do Temporary Workers More Often Decide to Work While Sick? Evidence for the Link between Employment Contract and Presenteeism in Europe

**DOI:** 10.3390/ijerph16101868

**Published:** 2019-05-27

**Authors:** Marvin Reuter, Morten Wahrendorf, Cristina Di Tecco, Tahira M. Probst, Sascha Ruhle, Valerio Ghezzi, Claudio Barbaranelli, Sergio Iavicoli, Nico Dragano

**Affiliations:** 1 Institute of Medical Sociology, Centre for Health and Society, Medical Faculty, University of Duesseldorf, 40225 Duesseldorf, Germany; wahrendorf@uni-duesseldorf.de (M.W.); dragano@med.uni-duesseldorf.de (N.D.); 2 Italian Workers’ Compensation Authority (INAIL), Department of Occupational and Environmental Medicine, Epidemiology and Hygiene, 00078 Monte Porzio Catone Rome, Italy; c.ditecco@inail.it (C.D.T.); s.iavicoli@inail.it (S.I.); 3 Department of Psychology, Washington State University, Vancouver, WA 98686, USA; probst@wsu.edu; 4 Chair of Business Administration, in particular Work, Human Resource Management and Organization Studies, Faculty of Business Administration and Economics, University of Duesseldorf, 40225 Duesseldorf, Germany; sascha.ruhle@uni-duesseldorf.de; 5 Department of Psychology, Sapienza University of Rome, 00185 Rome, Italy; valerio.ghezzi@uniroma1.it (V.G.); claudio.barbaranelli@uniroma1.it (C.B.)

**Keywords:** employment contract, temporary work, sickness presenteeism, presenteeism propensity, sickness presence, job insecurity, young workers

## Abstract

European employees are increasingly likely to work in cases of illness (sickness presenteeism, SP). Past studies found inconsistent evidence for the assumption that temporary workers decide to avoid taking sick leave due to job insecurity. A new measure to identify decision-based determinants of SP is presenteeism propensity (PP), which is the number of days worked while ill in relation to the sum of days worked while ill and days taken sickness absence. We investigated the link between employment contract and PP using cross-sectional data from 20,240 employees participating in the 2015 European Working Conditions Survey. Workers were grouped by type and duration of employment contract. The link between contract and PP was estimated using a multilevel Poisson model adjusted for socio-demographical, occupational and health-related covariates. We found that European employees worked 39% of the days they were ill. In contrast to previous studies, temporary workers were significantly more likely to decide for presenteeism than permanent workers were, especially when the contract was limited to less than 1 year. Controlling for perceived job insecurity did just marginally attenuate this association. Presenteeism was also more common among young and middle-aged workers; however, we did not find a significant interaction between contract and age affecting presenteeism. In conclusion, the employment contract is an important determinant of presenteeism. Our results give reason to believe that temporary workers show increased attendance behavior independent of job insecurity, because they are less likely to have access to social protection in case of illness.

## 1. Introduction

### 1.1. Background

Sickness presenteeism (SP), defined as going to work despite being ill, has gained increased attention during the last years [1,2]. Besides the fact that SP can lead to costs that even exceed those of sickness absence (SA) [3], it can be linked to reduced productivity [4] as well as to increased likelihoods for subsequent illness [5] and SA [6,7]. Frequent SP is also related to physical and mental health problems and elevated risks for future myocardial infarction or fatal coronary heart disease [8]. Additionally, employees working while ill can be contagious and infect other people in the work setting [9]. Nevertheless, opting for SP seems to be a common work behavior. Although most studies stem from Scandinavian countries and differ in their survey instruments used, on average 50–70% of respondents report working while sick at least once during a year [10,11,12,13]. Data from the 2010 European Working Conditions Survey show a prevalence of 40% in 34 countries [14]. A monitoring study from the UK found SP substantially increasing during the last years [15].

### 1.2. Temporary Employment and Presenteeism

SP is related to a range of socio-demographic and occupational factors. For example, studies found increased prevalence of SP among women, young and middle-aged workers, higher occupational positions and health care workers [16]. SP can also be related to absence policies, elevated job demands, job stress, low resources, discrimination and job attitudes [1]. Furthermore, studies indicate that SP is more often reported if workers face personnel cutbacks [12], downsizing [17,18] or a poor financial situation [13]. In the case of working contract, though, the evidence is less conclusive and studies that investigated if SP is more common among temporary workers than among workers with a permanent working contract report heterogeneous findings. The main assumption, hereby, is that workers with a temporary working contract may be more prone to SP, possibly as a mean to maintain their job or to get a permanent contract [2]. This assumption, though, has only received limited support, and most studies found no association between type of working contract and SP so far [1,11,13], with some studies even reporting lower SP rates among temporary workers compared with permanent workers [12,19,20]. Only one study with South Korean employees found temporary employment to be positively related to SP [21]. 

One reason for these inconsistent findings in the case of working contract may lie in the methodological approach used. As pointed out by Gerich, SP involves both a health process and a decision process [22]. The health process refers to the vulnerability for SP determined by factors that directly or indirectly affect a person’s health, for example, age, socio-economic position or work stress. The decision process, in contrast, refers to factors determining whether a person chooses to go to work in case of illness or to stay at home. Health processes and decision processes can both determine the SP rate. For example, in case a study found that older workers have a SP rate of 10 days a year and young workers only 5. Then it would misleading to conclude that older workers work twice as often during illness, because higher SP rates may simply be due to poorer health status of older workers. In fact, there is robust evidence that higher SA rates are associated with higher SP rates [11,12,13,18,23]. Therefore, investigating if temporary employment and presenteeism are linked together is difficult by just looking at SP rates. One reason is that temporary workers generally show lower SA rates than permanent workers [20,24]. A possible explanation is that temporary workers are more likely to end up unemployed following periods of high SA rates, because they are less protected by their contract than permanent workers are (healthy worker effect) [25].

As proposed by Gerich, a more appropriate method to analyze decision-based determinants of SP is given by the presenteeism propensity (PP) [22]. The PP indicates the days worked while ill in relation to the overall number of health events, which is approximately the sum of SP days and SA days. In other words, the PP allows investigating the decision for presenteeism independent of health, considering each day the individual chooses between SP and SA as event. SP and PP have been compared in their relationship to potential determinants of presenteeism [26,27] and it was found that PP is a better approach to identify factors restricting the decision to SA.

### 1.3. Aims and Hypothesis

This study aims to analyze the link between employment contract and SP in a broad data set of European employees. Since we use a new approach that is more useful in identifying decision-based determinants of SP, our objective is to overcome the methodological limitations of past studies. Additionally, we analyze whether the relationship between employment contract and presenteeism differs between young and older workers. Young workers represent a group more often affected by adverse employment conditions as temporary working contracts [28], insecure employment [29] or limited access to occupational health and safety [30]. Since they are group of special risk, we hypothesize that the link between contract and SP could be differently pronounced among young workers. Taken together, this study tests the following hypotheses:

**Hypothesis 1** **(H1).** *Employees with a temporary employment contract demonstrate a higher presenteeism propensity than those having a permanent contract*.

**Hypothesis 2** **(H2).** *The link between employment contract and presenteeism propensity depends on a worker’s age*.

## 2. Materials and Methods

### 2.1. Data

Data were used from Round 6 (2015) of the European Working Conditions Survey [31]. The EWCS is a repeated cross-sectional study conducted by the European Foundation for the Improvement of Living and Working Conditions (Eurofound). Since 1991 the EWCS has collected data on the working conditions of the population in 36 European countries. Study participants were 15 years or older and worked for pay or profit for at least one hour per week following the definition of the International Labour Organization (ILO). Participants were selected by drawing a multi-stage, stratified, random sample in each country. Sample sizes ranged from 1000 to 3300 cases per country. Face to face interviews took place at the respondents’ homes between February and September 2015. The average response rate was 43%. A more detailed description of the methodology can be found in the technical report [32]. 

### 2.2. Study Sample

We reduced the initial sample of 43,850 participants to employees and excluded self-employed workers, and individuals being unemployed, retired, in full-time education or unable to work due to long-term illness or disability at the time of the survey, which led to *N* = 32,392 observation (73.9% of the initial sample). We also restricted the sample to respondents that were between 15–65 years old and working a minimum of 10h/week, leading to a sample of *N* = 31,300 (71.4%). To investigate only the behavior of employees without chronic diseases, we omitted cases reporting high numbers of SA or SP of more than 70 days during the last year. This cut-off was chosen in accordance to previous studies [33]. This led to a sample of *N* = 30,943 (70.6%) employees from 33 European countries. Moreover, because PP can only be calculated for employees who reported at least one health event (either SA or SP), we excluded individuals neither reporting days of SA nor days of SP. This further reduced the sample to *N* = 20,240 (46.2%). Respondents working for less than one year in their job (*n* = 1,789; 8.8%) were not precluded, but analyses were controlled for job tenure.

### 2.3. Variables

#### 2.3.1. Sickness Absenteeism, Sickness Presenteeism, Health Events and Presenteeism Propensity

Sickness absenteeism (SA) was measured by the question “Over the past 12 months (or since you started your main paid job), how many days in total were you absent from work due to sick leave or health-related leave?” Respondents could directly state the number of working days. 

Sickness presenteeism (SP) was measured by the questions: “Over the past 12 months or since you started your main paid job, did you work when you were sick?” If the answer was “yes” respondents were asked to state the number of days working while sick in an open-ended response format. We allocated zero SP days if respondents were not working when they were sick. 

Following the definition of Gerich, the number of health events (HE) was calculated as the sum of SP and SA days [22]. The individual presenteeism propensity (PP) was calculated as the ratio between SP days and the sum of SP and SA days. Therefore, PP could range between 0 (No day worked while sick) and 1 (Worked on each day during sickness):

(1) $$Presenteeism\;propensity\;\left(PP\right)=\frac{SP\;days}{Health\;events}\approx \frac{SP\;days}{\left(SP\;days+SA\;days\right)}$$

**Figure A1:**
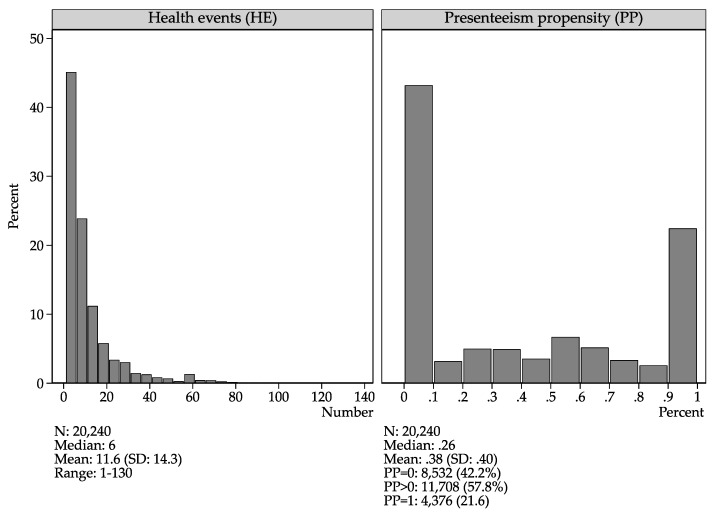
Distribution of health events and presenteeism propensity in the EWCS 2015.

Presenteeism propensity was not normally distributed and the two most frequent values were working 0% or 100% while ill (see Appendix A, Figure A1).

#### 2.3.2. Employment Contract and Job Insecurity

Respondents were categorized according to their type of working contract in having a contract of unlimited duration, having a temporary contract or having no contract or “other”. No contracts were employment relations without formal written contracts (oral contracts). Those having a temporary contract were further distinguished by their contract duration between ≥1 year and less than one year. This was to account for the fact that individuals with a short-term contract might experience more insecurity compared to those with a long-term contract.

In order to examine whether the link between temporary employment and presenteeism could be explained by anticipated job loss, we also included an item concerning perceived job insecurity. Job insecurity was assessed by the statement “I might lose my job in the next 6 months” followed by a five-point Likert scale including the answers “strongly agree”, “tend to agree”, “neither agree nor disagree”, “tend to disagree” and “strongly disagree”. Respondents were classified as perceiving job insecurity when strongly agreed or tended to agree.

#### 2.3.3. Socio-Demography and Occupational Factors

We considered socio-demographic and job-related confounders including sex, age, occupational position, working sector, company size, job tenure, weekly working hours and income as these were factors found to be linked to SP [1,16]. As a measure of occupational position, we regrouped occupations according to the European Socio-economic Classification (ESeC) scheme into four categories and the classification of working sector was based on the *Nomenclature statistique des activités économiques dans la Communauté européenne* (NACE). Company size was measured by the number of employees working in the organization (“<10”, “10–249” or “250+”). The monthly net income from the main paid job in Euro was divided by the country-specific median and expressed in percent. Respondent’s age, job tenure and weekly working hours were measured as continuous variables. In order to conduct analyses stratified by age, we grouped participants into those being 15–29 years, 30–49 years and 50–65 years old. If respondents had worked for less than one year in the organization, we coded job tenure as “0.” 

#### 2.3.4. Handling of Missing Values

Missing values were found in every of the 12 variables ranging between 0.02–12.66% (see Appendix A, Table A1). Of the 32,392 observations 22,740 (70.2%) were complete cases without missing values. There were 8,051 (24.9%) observations with missing values in only 1 variable and 1,601 (4.9%) observations with missing in more than one variable. Patterns of missing values were non-monotone.

Little’s MCAR test showed a χ^2^-distance of 3438.9 with degrees of freedom = 1868 (*p* < 0.001). The test provides evidence that missing data in the 12 variables of interest are not MCAR (missing completely at random). Thus, complete case analysis would lead to biased estimates [34] and we therefore filled missing values by using chained multiple imputation [35]. Multiple imputation was conducted using Stata’s “mi impute chained” procedure and repeated five times with 10 iterations, respectively. Estimation results were pooled. 

### 2.4. Statistical Analyses

We described the study population in terms of socio-demographic, occupational and health-related characteristics and compared the mean PP by these factors among young, middle-aged and old workers. Since we use a cross-country data set, we visualized the PP along European countries using a choropleth map. The mean country PP was adjusted for gender, age, job tenure, weekly working hours and working sector to allow comparing populations with different socio-demographical and labor market structures. 

We applied a series of multilevel Poisson regression models to examine the relationship between employment contract and PP. As the distribution of PP (see Appendix A, Figure A1) is difficult to predict by linear regression, we first used a generalized linear model with a binomial distribution and a log link function [36]. However, due to failed convergence we decided for a multilevel Poisson regression with robust variance estimation [37]. We calculated rate ratios (RR) to compare the PP between groups and by continuous variables. Since we used a country data set, we decided for a multi-level model to account for between-country variance. The Median Rate Ratio (MRR) indicates the level of heterogeneity in the outcome attributable to country differences. The MRR indicates the average change in the RR when comparing two identical subjects from two randomly selected different countries that are ordered by their PP rate [38]. The MRR indicates the average RR when comparing a low PP country to a high PP country. 

To describe the link between employment contract and PP among different age groups, we conducted a first regression analysis separately for young, middle-aged and older workers.

To test our hypotheses, we applied a hierarchical regression analysis taken all age groups together. In a first step, unadjusted estimators for PP by employment contract were calculated to examine if temporary workers demonstrate a higher PP than permanent workers (Hypothesis 1). In Model 1, socio-demographical and occupational covariates as well as the number of health events were included, to test for confounding. In Model 2, we included perceived job insecurity to investigate whether the relationship between contract and PP was robust against job insecurity. To determine whether the link between contract and presenteeism varies significantly between young and older workers, Model 3 additionally includes an interaction term between contract and age (Hypothesis 2). A Wald test was used to determine whether the interaction explained a significant part of the variation of PP. A Wald test is preferable to a Likelihood ratio test in cases of multilevel models with robust standard errors [39]. To account for the fact that workers with only a few health events can vary in their decision to presenteeism from workers with more health events [22,40], models also include a dummy variable indicating whether the PP was based on 1-9 health events or more. Continuous variables such as job tenure, weekly working hours and income were standardized. We used quadratic terms to test for non-linear relationships between continuous predictors and the outcome. All analyses were performed using Stata 15.1 MP (64-bit, StataCorp LLC, College Station, TX, USA).

## 3. Results

### 3.1. Sample Description

Table 1 shows the study population by socio-demographic, occupational and health-related variables. We excluded 10,703 (34.6%) employees that had not experienced any health event during the last year. Within this group, we found participants more often being younger, male, having a lower job tenure, lower occupational positon and more often working under a non-permanent working contract compared to those included in the study. The sample used for the following analyses consisted of 20,240 employees that had experienced at least one health event (SA or SP) during the last year. Within this group, around 20% had a non-permanent working contract and 25% of those contracts had a duration of less than one year. The mean number of health events leading to either SA or SP was 11.6 (±14.3). The mean PP was 0.39 (±0.41), indicating that European employees worked on 39% of the days they were ill. 57.8% had worked at least on one day during sickness, 35.4% on more than half of the days and 21.6% during all days of sickness.

### 3.2. Patterns of Presenteeism

Table 2 shows mean PP along socio-demographic and occupational covariates for young, middle-aged and old workers. We found PP higher among young and middle-aged workers compared to the oldest cohort. In all three age groups, temporary workers were more likely to exhibit presenteeism than permanent workers were. Employees with a contract duration of less than one year were the group demonstrating the highest PP. Having no formal working contract was not related to increased PP. Further factors positively associated with PP were job insecurity, female gender, high occupational position and working in a large company. We found also a U-shaped association between PP and working hours, job tenure and income, despite some deviations for this among young workers. SP was less common in manual jobs (as in agriculture, industry, construction and transport sectors) and more common in service-related sectors (financial, education and health). Regardless of age, PP was lower when the number of health events was low.

Table 3 shows the likelihood of PP by type of working contract among different age groups adjusted for country and socio-demographic and occupational factors. In all three age groups, workers with a temporary contract decided more often for presenteeism than workers with a permanent contract. This relationship was statistically significant in all three age groups. However, among young workers, having a long-term temporary contract did not reach statistical significance.

**Figure 1:**
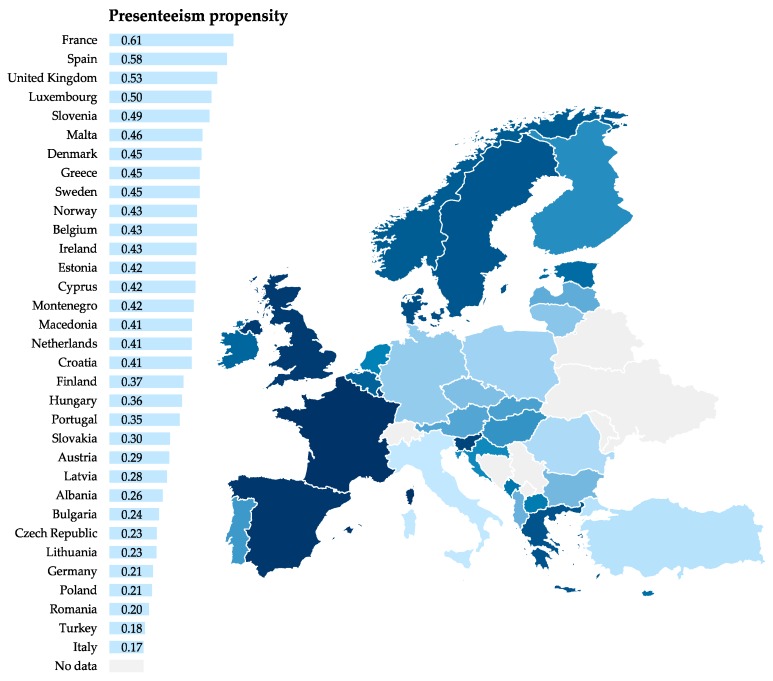
Means of presenteeism propensity in 33 European countries (2015) adjusted for sex, age, job tenure, working hours, occupation and working sector.

Figure 1 shows the mean PP between European countries. Propensities varied widely but no clear pattern was observable. Presenteeism was a more common work behavior in France, Spain, United Kingdom and in Scandinavian countries and in comparison to that more rarely in Germany, Poland, Romania, Turkey and Italy. We observed no significant correlation between temporary work (long or short-term) and PP at the country-level (*r* = 0.066, *p* = 0.713). 

### 3.3. Employment Contract and Likelihood for Presenteeism

Table 4 depicts the results of the multilevel Poisson regression models. The MRR was 1.38, indicating that the median increase of PP was 1.38 between a low PP and a high PP country when individual characteristics were hold constant. As shown by the unadjusted results, temporary workers opted more often for SP than permanent workers did. After adjusting for socio-demographic, occupational factors and health events in Model 1, this statistical relationship remained constant and was not attenuated. This confirms our first hypothesis (H1). Temporary workers decided 1.11 times more often to work during illness than permanent workers (*p* < 0.01). When the contract was limited to less than 1 year, the differences was even higher (RR = 1.29, *p* < 0.001). Employees without a formal contract tended also more often to presenteeism than permanent workers, but the difference was not significant. 

In Model 2, we tested whether perceived job insecurity explained the relationship between contract and presenteeism. Employees fearing job loss were more likely to work while sick (RR = 1.09, *p* < 0.01). However, after controlling for job insecurity, the relationship between employment contract and presenteeism was just partly attenuated and remained significant, indicating that temporary workers tend more often to presenteeism independent of job insecurity. 

In Model 3 we tested whether the link between contract and presenteeism varied by age. Although we found a trend towards an higher association between contract and PP among older age groups compared to young workers, the overall interaction term did not significantly explain any additional variance (Wald test: *p* = 0.129). Therefore, we had to reject our second hypothesis (H2).

Additionally, we observed job tenure and weekly working hours positively correlated with presenteeism. Managers and professionals were more likely to work while sick compared to blue-collar workers. PP was also higher in jobs related to the educational sector. A high number of health events was linked to a lower PP. No significant link to PP was found by income and company size. We also found that the variation in RR between countries was greater than by each of the individual-level characteristics. This indicates country as a very important determinant of presenteeism (see Appendix A, Table A2).

**Figure 2:**
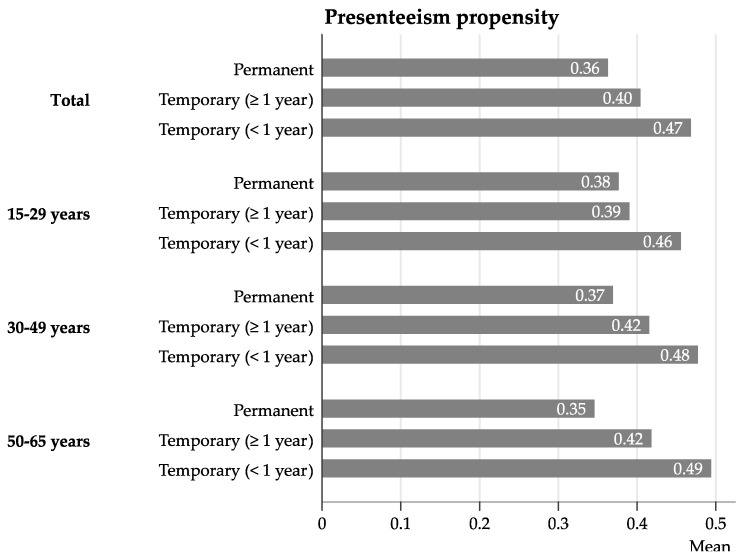
Predicted presenteeism propensity by employment contract in different age groups. Propensities adjusted for country, sex, job tenure, working hours, income, occupation, working sector, company size and number of health events.

Figure 2 shows the predicted PP by type of employment contract adjusted for covariates. Regardless of age, temporary workers decided more often to work in cases of illness than permanent workers. The difference was higher when the contractual duration was less than 1 year. Employees with a permanent employment contract worked on 36% of the days they were ill. In contrast, employees having a contract limited to less than one year worked 47% of the days. This gap seems to be higher for older workers; however, this interaction was not significant. 

### 3.4. Sensitivity Analysis

Additional models were calculated to analyze how results varied when dichotomizing PP by different cut-offs (see Appendix A, Table A3). Temporary employment was significantly associated to presenteeism regardless whether we defined it by a PP > 0 (presenteeism ever) or PP = 1 (all days worked during sickness). However, the link between temporary work and presenteeism was stronger the higher the cut-off was. This indicates temporary workers not only having a higher likelihood to work while ill compared to permanent workers, but also working on more days during illness.

## 4. Discussion

The aim of this article was twofold: First, to investigate whether temporary employment increases the tendency to presenteeism in a representative sample of European workers. Second, to examine if the link between temporary employment and presenteeism varies between young, middle-aged and older workers. While we could show that temporary workers were more likely to decide for presenteeism, we found this association equally between young, middle-aged and older workers and not significantly depending on age.

### 4.1. General Patterns of Presenteeism

To our knowledge, this is the first study presenting data about PP in a large European dataset with 33 countries. In total, 65.4% of the European workers reported at least one health event (SA or SP) during the last 12 months. Within this group, the mean PP was 0.39 (±0.41), indicating that employees went to work on averagely 39% of the days they were ill. This shows that European employees very often opt for presenteeism in cases of sickness. However, other studies even observed higher numbers. For example, Biron et al. found a PP of 51.5% in a sample of 3825 Canadian employees [40], whereas Gerich observed a PP of 59% in a sample of 781 Austrian employees [26]. However, these differences seem easily explainable because our sample includes countries where we have found presenteeism to be a less common work behavior. We also found presenteeism more common among young and middle-aged workers, as well as among women, managers, professionals, and workers with long working hours. This was in line with previous findings [16]. 

We also observed that PP varied widely between European countries ranging from 0.17 to 0.61. Since this is the first study presenting data on PP along European countries, comparisons to previous findings are not possible. Varying presenteeism tendency could be attributable to cultural or legal differences between countries in terms of working norms, absence policies or the generosity of paid sick leave as these are factors associated with attendance behavior. This calls for future analyses of contextual factors determining presenteeism, as already proposed by Johns [41]. 

### 4.2. The Relationship between Employment Contract and Presenteeism

With respect to our first hypothesis, we found temporary employment increasing the likelihood of choosing presenteeism. Most of the previous studies have found temporary employment not associated with presenteeism or even negatively correlated. As already discussed in the introduction, past studies that just compared SP days did actually not focus on the decision process. Our results clearly suggest that decision-based determinants of presenteeism should be investigated using PP instead of SP days to separate the effects of health and decision.

Further, we found that temporary workers engaged in presenteeism in response to sickness more often than permanent employees did. One explanation may point to possible health disparities between permanent and temporary workers; for example, workers with severe chronic or relapsing health conditions may be less likely to gain permanent employment. However, we excluded all study participants with chronic health conditions or high SA or SP rates from this study and adjusted analyses for the number of health events. Therefore, another explanation for our findings may be that employees in non-permanent settings feel increased pressure or obligation to show up at work during illness in order to maintain their job or to increase their chances for permanent employment in the future [1]. However, we tested this explanation by controlling for perceived job insecurity and despite the link between contract and PP was attenuated there remained an association that was still highly significant. We therefore suppose other factors restricting the decision of temporary workers to SA. For example, European countries differently regulate entitlements to paid sick leave and sickness benefits [42]. Caused by shortened contribution periods of temporary workers [28], they have less likely access to occupational safety and health services and to social protection in case of illness compared to permanent employees and therefore do more often decide for presenteeism independent of job insecurity. 

### 4.3. Strengths and Limitations

This study adds evidence to the existing knowledge about occupational determinants of SP. By using PP instead of SP frequency, several limitations of past studies could be overcome. This included focusing on the decision processes and excluding healthy workers that did not need to make any decisions for or against presenteeism. We were also able to show that the duration of the working contract does matter, whereas age context plays a lesser role. Because we used multiple imputation to fill missing values, we could prevent estimates from being biased. On the other side, some limitations regarding the cross-sectional character of the study design have to be mentioned. Although we controlled for a wide range of confounders, the results must be interpreted cautiously in terms of drawing any causal inferences. Finally, as with any self-reported measures, responses to SA and SP may suffer from recall bias. 

## 5. Conclusions

To conclude, having a permanent or non-permanent job position is an important determinant for a workers decision to come to work during illness or take a sick leave. In particular, employees with short-term contracts of less than one year may be at greater risk for presenteeism. Future studies could investigate whether the length of contribution periods on country-level can explain differences in presenteeism between temporary and permanent workers. Prevention measures of presenteeism could include paid sick leave policy or absence management policies that focus for example on standard operating procedures in cases of sickness. On country-level, legal changes towards shortened contribution periods have the potential to improve access to social protection in case of sickness. Supported by our findings, effectiveness of those measures could be improved when especially focusing on temporary workers. Furthermore, our findings strongly suggest PP as a superior measure to investigate determinants of presenteeism.

## Figures and Tables

**Table 1:** Study population by socio-demographic, occupational and health-related characteristics.

Variable	Categories or Range	In Study (≥1 Health Event)		Not in Study (No Health Event)	
		*N*/(Mean)	%/(±SD)	*N*/(Mean)	%/(±SD)
**Type of working contract**	Permanent	16,529	81.7	7978	74.5
	Temporary (≥1 year)	1808	8.9	1189	11.1
	Temporary (<1 year)	480	2.4	576	5.4
	No contract/other	1423	7.0	960	9.0
**Perceived job insecurity**	No	16,985	83.9	8935	83.5
	Yes	3255	16.1	1768	16.5
**Sex**	Men	9439	46.6	5596	52.3
	Women	10,801	53.4	5107	47.7
**Age**	15–29 years	3267	16.1	2126	19.9
	30–49 years	10,802	53.4	5394	50.4
	50–65 years	6171	30.5	3183	29.7
**Job tenure (years)**	0–50	(10.0)	(±9.4)	(8.9)	(±9.6)
**Weekly working hours**	10–105	(38.8)	(±9.7)	(38.5)	(±10.1)
**Income (% of country median)**	1–2750	(121.6)	(±75.1)	(119.8)	(±75.0)
**Occupational position (ESeC)**	Blue-collar workers	5311	26.2	3308	30.9
	White-collar workers	4236	20.9	2576	24.1
	Intermediates, low. supervisory	2818	13.9	1367	12.8
	Managers and professionals	7875	38.9	3452	32.3
**Working sector (NACE)**	Agriculture	309	1.5	251	2.3
	Industry	3428	16.9	1910	17.8
	Construction	1204	5.9	736	6.9
	Transport	1212	6.0	687	6.4
	Commerce and hospitality	3748	18.5	2353	22.0
	Financial services	812	4.0	292	2.7
	Other services	3458	17.1	1812	16.9
	Public administration	1439	7.1	698	6.5
	Education	2180	10.8	963	9.0
	Health	2450	12.1	1001	9.4
**Company size**	<10 employees	5505	27.2	3658	34.2
	10–249 employees	11,263	55.6	5690	53.2
	250+ employees	3472	17.2	1355	12.7
**Sickness absenteeism (SA)**	0–70 days	(7.4)	(±11.2)	(0.0)	(±0.0)
**Sickness presenteeism (SP)**	0–70 days	(4.2)	(±7.5)	(0.0)	(±0.0)
**Health events (HE = SA + SP)**	1–130 days	(11.6)	(±14.3)	(0.0)	(±0.0)
**Presenteeism propensity (PP = SP/HE)**	0-1	(0.39)	(±0.41)		
	PP = 0	8532	42.2		
	PP > 0	11,708	57.8		
	PP > 0.50	7167	35.4		
	PP = 1	4376	21.6		
Sample size		20,240	100.0	10,703	100.0

SD = Standard deviation. SA and SP related to the time span of the last 12 months.

**Table 2:** Prevalence of covariates and means of presenteeism propensity in different age groups.

Variable	15–29 Years *N* = 3267			30–49 Years *N* = 10,802			50–65 Years *N* = 6171		
	%	Mean	SD	%	Mean	SD	%	Mean	SD
**Type of working contract**									
Permanent	65.1	0.37	±0.40	83.3	0.39	±0.40	87.7	0.37	±0.40
Temporary (≥1 year)	17.7	0.40	±0.40	8.4	0.42	±0.42	5.3	0.39	±0.42
Temporary (<1 year)	4.6	0.54	±0.43	2.4	0.58	±0.43	1.2	0.55	±0.44
No contract/other	12.7	0.36	±0.40	6.0	0.37	±0.41	5.8	0.39	±.040
**Perceived job insecurity**									
No	81.5	0.37	±0.40	84.4	0.39	±0.40	84.5	0.37	±0.41
Yes	18.5	0.44	±0.40	15.7	0.43	±0.41	15.5	0.38	±0.41
**Sex**									
Men	49.3	0.37	±0.40	46.6	0.38	±0.41	45.3	0.35	±0.41
Women	50.7	0.40	±0.40	53.4	0.41	±0.40	54.7	0.39	±0.41
**Job tenure**									
<1 year	23.7	0.39	±0.42	7.5	0.45	±0.44	3.4	0.38	±0.43
1–10 years	75.5	0.39	±0.40	61.3	0.38	±0.40	35.4	0.36	±0.40
>10 years	0.9	0.32	±0.42	31.2	0.42	±0.41	61.2	0.38	±0.41
**Weekly working hours**									
10–24 h	11.0	0.47	±0.43	8.4	0.46	±0.42	10.4	0.41	±0.42
25–39 h	27.1	0.39	±0.40	28.7	0.40	±0.39	31.7	0.36	±0.39
40–54 h	54.9	0.36	±0.39	57.3	0.38	±0.41	54.1	0.36	±0.41
55+ hours	7.0	0.42	±0.43	5.6	0.49	±0.42	3.8	0.52	±0.43
**Income (% of country median)**									
<60%	14.2	0.44	±0.42	7.1	0.47	±0.43	8.1	0.43	±0.43
60–99%	41.2	0.38	±0.40	28.8	0.39	±0.41	29.0	0.36	±0.40
100–150%	32.4	0.39	±0.40	41.4	0.39	±0.40	39.7	0.35	±0.40
>150%	12.2	0.35	±0.40	22.7	0.40	±0.41	23.3	0.40	±0.42
**Occupational position (ESeC)**									
Blue-collar workers	23.9	0.35	±0.40	25.5	0.36	±0.41	28.7	0.31	±0.40
White-collar workers	30.3	0.38	±0.41	19.5	0.37	±0.41	18.5	0.38	±0.40
Intermediates and lower supervisory	14.0	0.39	±0.41	14.1	0.38	±0.41	13.6	0.35	±0.40
Managers and professionals	31.8	0.42	±0.40	40.9	0.43	±0.40	39.2	0.42	±0.41
**Working sector (NACE)**									
Agriculture	1.5	0.40	±0.41	1.4	0.34	±0.40	1.8	0.32	±0.40
Industry	15.1	0.35	±0.40	17.3	0.37	±0.41	17.3	0.34	±0.40
Construction	6.2	0.33	±0.39	5.8	0.36	±0.40	6.1	0.34	±0.41
Transport	4.6	0.37	±0.40	6.1	0.39	±0.41	6.6	0.32	±0.40
Commerce and hospitality	28.5	0.40	±0.42	18.4	0.39	±0.42	13.5	0.36	±0.41
Financial services	3.2	0.48	±0.38	4.4	0.39	±0.40	3.8	0.43	±0.43
Other services	20.7	0.38	±0.40	17.3	0.41	±0.41	14.8	0.38	±0.40
Public administration	4.4	0.30	±0.36	7.1	0.41	±0.39	8.5	0.40	±0.42
Education	6.8	0.43	±0.39	10.5	0.45	±0.40	13.3	0.41	±0.41
Health	9.1	0.44	±0.39	11.8	0.41	±0.40	14.3	0.39	±0.39
**Company size**									
<10 employees	35.2	0.37	±0.42	26.6	0.39	±0.42	24.1	0.36	±0.42
10–249 employees	51.1	0.39	±0.40	55.7	0.39	±0.40	57.9	0.37	±0.40
250+ employees	13.7	0.40	±0.39	17.7	0.42	±0.39	18.0	0.40	±0.40
**Number of health events**									
1–9	69.5	0.39	±0.43	62.3	0.41	±0.43	56.4	0.40	±0.44
10+	30.6	0.37	±0.35	37.7	0.38	±0.35	43.6	0.34	±0.36
Total	100.0	0.39	±0.40	100.0	0.40	±0.41	100.0	0.37	±0.41

**Table 3:** Rate ratios (RR) of presenteeism propensity by employment contract and age.

Variable	15–29 Years		30–49 Years		50–65 Years	
	RR	95% CI	RR	95% CI	RR	95% CI
**Type of working contract**						
Permanent	Reference		Reference		Reference	
Temporary (≥1 year)	1.04	[0.94–1.15]	1.12 **	[1.03–1.21]	1.18 **	[1.05–1.33]
Temporary (<1 year)	1.27 **	[1.07–1.51]	1.29 ***	[1.16–1.42]	1.41 **	[1.14–1.74]
No contract/other	1.00	[0.83–1.19]	1.06	[0.96–1.16]	1.12	[1.00–1.26]
Individuals	3267		10,802		6171	
Countries	33		33		33	

Adjusted for country, sex, job tenure, working hours, income, occupation, working sector, company size and number of health events. * *p* < 0.05, ** *p* < 0.01, *** *p* < 0.001.

**Table 4:** Multilevel Poisson regression: Rate ratios (RR) of presenteeism propensity by different sets of covariates.

Variable	Unadjusted		M1		M2		M3	
	RR	95% CI	RR	95% CI	RR	95% CI	RR	95% CI
**Type of working contract**								
Permanent	Reference		Reference		Reference		Reference	
Temporary (≥1 year)	1.11 **	[1.03–1.18]	1.11 ***	[1.05–1.18]	1.09 **	[1.03–1.16]	1.02	[0.92–1.12]
Temporary (<1 year)	1.28 ***	[1.15–1.41]	1.29 ***	[1.17–1.42]	1.24 ***	[1.13–1.37]	1.16 *	[1.01–1.34]
No contract/other	1.09	[0.98–1.21]	1.07	[0.97–1.19]	1.07	[0.97–1.18]	1.01	[0.86–1.20]
**Age**								
15–29 years			Reference		Reference		Reference	
30–49 years			1.01	[0.96–1.05]	1.00	[0.96–1.05]	0.98	[0.93–1.03]
50–65 years			0.95	[0.90–1.02]	0.95	[0.89–1.01]	0.91 **	[0.85–0.98]
**Perceived job insecurity**								
Yes					1.09 **	[1.03–1.15]	1.09 **	[1.03–1.15]
**Interaction contract x age**								
Temporary (≥1 year) x 30–49 years							1.09	[0.98–1.21]
Temporary (≥1 year) x 50–65 years							1.17 *	[1.03–1.33]
Temporary (<1 year) x 30–49 years							1.08	[0.95–1.23]
Temporary (<1 year) x 50–65 years							1.18	[0.95–1.47]
No contract/other x 30–49 years							1.04	[0.92–1.18]
No contract/other x 50–65 years							1.11	[0.96–1.30]
**(Intercept)**	0.34 ***	[0.30–0.39]	0.31 ***	[0.26–0.38]	0.31 ***	[0.26–0.38]	0.32 ***	[0.26–0.39]
**Median Rate Ratio (MRR)**	1.38		1.38		1.39		1.39	
**Model information**								
−2logpseudolikelihood	28,325.3		28,180.5		28,173.5		28,170.7	
Deviance (%)	−0.07		−0.59		−0.61		−0.62	
Wald test	*p* < 0.001		*p* < 0.001		*p* = 0.002		*p* = 0.129	
Individuals	20,240		20,240		20,240		20,240	
Countries	33		33		33		33	

M1 adjusted for sex, job tenure, working hours, income, occupation, working sector, company size and number of health events. M2 additionally included perceived job insecurity. M3 additionally included interaction between contract and age. * *p* < 0.05, ** *p* < 0.01, *** *p* < 0.001.

## Appendix A

**Table A1:** Absolute and relative frequencies of missing values before imputation.

No	Variable	Name	Missing (*N*)	Total (*N*)	Missing (%)
(1)	female	Female	6	32,392	0.02
(2)	age	Age	112	32,392	0.35
(3)	jobten2	Job tenure	419	32,392	1.29
(4)	wrkhours	Weekly working hours	562	32,392	1.73
(5)	nace_long	Working sector (NACE)	155	32,392	0.48
(6)	esec4	Occupational position (ESeC)	136	32,392	0.42
(7)	estm	Company size	678	32,392	2.09
(8)	contract2	Employment contract	77	32,392	0.24
(9)	jobinsec2	Perceived job insecurity	2607	32,392	8.05
(10)	jobinc	Income (% of country median)	4101	32,392	12.66
(11)	sickab	Days of sickness absenteeism	3334	32,392	10.29
(12)	sickpr	Days of sickness presenteeism	1339	32,392	4.13

**Table A2:** Multilevel Poisson regression: Rate ratios (RR) of presenteeism propensity by different sets of covariates (Complete version of Table 4).

Variable	Unadjusted		M1		M2		M3	
	RR	95% CI	RR	95% CI	RR	95% CI	RR	95% CI
**Type of working contract**								
Permanent	Reference		Reference		Reference		Reference	
Temporary (≥1 year)	1.11 **	[1.03–1.18]	1.11 ***	[1.05–1.18]	1.09 **	[1.03–1.16]	1.02	[0.92–1.12]
Temporary (<1 year)	1.28 ***	[1.15–1.41]	1.29 ***	[1.17–1.42]	1.24 ***	[1.13–1.37]	1.16 *	[1.01–1.34]
No contract/other	1.09	[0.98–1.21]	1.07	[0.97–1.19]	1.07	[0.97–1.18]	1.01	[0.86–1.20]
**Sex**								
Women			1.08 ***	[1.04–1.12]	1.08 ***	[1.05–1.12]	1.08 ***	[1.05–1.12]
**Age**								
15–29 years			Reference		Reference		Reference	
30–9 years			1.01	[0.96–1.05]	1.00	[0.96–1.05]	0.98	[0.93–1.03]
50–65 years			0.95	[0.90–1.02]	0.95	[0.89–1.01]	0.91 **	[0.85–0.98]
**Job tenure**			1.03 *	[1.00–1.06]	1.04 *	[1.01–1.06]	1.04 **	[1.01–1.07]
**Job tenure ^2^**			1.00	[0.99–1.01]	1.00	[0.99–1.01]	1.00	[0.99–1.01]
**Weekly working hours**			1.07 ***	[1.04–1.09]	1.07 ***	[1.04–1.09]	1.07 ***	[1.04–1.09]
**Weekly working hours ^2^**			1.03 ***	[1.03–1.04]	1.03 ***	[1.03–1.04]	1.03 ***	[1.03–1.04]
**Income**			0.97	[0.94–1.01]	0.97	[0.94–1.01]	0.97	[0.94–1.01]
**Income ^2^**			1.00	[1.00–1.00]	1.00	[1.00–1.00]	1.00	[1.00–1.00]
**Occupational position**								
Blue-collar workers			Reference		Reference		Reference	
White-collar workers			1.04	[0.97–1.11]	1.04	[0.97–1.11]	1.04	[0.97–1.11]
Intermediates, low. supervisory			1.04	[0.97–1.12]	1.04	[0.97–1.12]	1.04	[0.97–1.12]
Managers and professionals			1.15 ***	[1.07–1.24]	1.15 ***	[1.07–1.24]	1.15 ***	[1.07–1.24]
**Working sector (NACE)**								
Agriculture			0.93	[0.79–1.10]	0.93	[0.80–1.10]	0.93	[0.79–1.10]
Industry			1.01	[0.95–1.08]	1.01	[0.95–1.07]	1.01	[0.95–1.07]
Construction			1.01	[0.93–1.09]	1.01	[0.93–1.09]	1.01	[0.93–1.09]
Transport			0.99	[0.90–1.08]	0.99	[0.90–1.08]	0.99	[0.90–1.08]
Commerce and hospitality			1.02	[0.97–1.08]	1.02	[0.97–1.08]	1.02	[0.97–1.08]
Financial services			1.02	[0.95–1.09]	1.02	[0.95–1.09]	1.02	[0.95–1.09]
Other services			Reference		Reference		Reference	
Public administration			1.01	[0.95–1.07]	1.01	[0.95–1.07]	1.01	[0.95–1.07]
Education			1.05 *	[1.00–1.11]	1.06 *	[1.01–1.11]	1.06 *	[1.01–1.11]
Health			0.97	[0.92–1.03]	0.98	[0.93–1.03]	0.98	[0.93–1.03]
**Company size**								
<10 employees			Reference		Reference		Reference	
10–249 employees			0.97	[0.92–1.01]	0.96	[0.92–1.01]	0.97	[0.92–1.01]
250+ employees			0.98	[0.92–1.04]	0.98	[0.92–1.04]	0.98	[0.92–1.04]
**Number of health events**								
10+			0.91 *	[0.85–0.98]	0.91 *	[0.85–0.98]	0.91 *	[0.85–0.98]
**Perceived job insecurity**								
Yes					1.09 **	[1.03–1.15]	1.09 **	[1.03–1.15]
**Interaction contract x age**								
Temporary (≥1 year) x 30–49 years							1.09	[0.98–1.21]
Temporary (≥1 year) x 50–65 years							1.17 *	[1.03–1.33]
Temporary (<1 year) x 30–49 years							1.08	[0.95–1.23]
Temporary (<1 year) x 50–65 years							1.18	[0.95–1.47]
No contract/other x 30–49 years							1.04	[0.92–1.18]
No contract/other x 50–65 years							1.11	[0.96–1.30]
**(Intercept)**	0.34 ***	[0.30–0.39]	0.31 ***	[0.26–0.38]	0.31 ***	[0.26–0.38]	0.32 ***	[0.26–0.39]
**Median Rate Ratio (MRR)**	1.38		1.38		1.39		1.39	
**Model information**								
−2logpseudolikelihood	28325.3		28180.5		28173.5		28170.7	
Deviance to Nullmodel (%)	−0.07		−0.59		−0.61		−0.62	
Wald test	*p* < 0.001		*p* < 0.001		*p* = 0.002		*p* = 0.129	
Individuals	20,240		20,240		20,240		20,240	
Countries	33		33		33		33	

* *p* < 0.05, ** *p* < 0.01, *** *p* < 0.001. ^2^ Quadratic term.

**Table A3:** Multilevel Poisson regression: Prevalence ratios (PR) for the likelihood of presenteeism propensity > 0 (presenteeism ever) and PP = 1 (every day worked while ill).

Variable	PP > 0 ^a^		PP > 0 ^a^		PP = 1 ^a^		PP = 1 ^a^	
	PR	95% CI	PR	95% CI	PR	95% CI	PR	95% CI
**Type of working contract**								
Permanent	Reference		Reference		Reference		Reference	
Temporary (≥1 year)	1.07 *	[1.00–1.15]	1.05	[0.93–1.19]	1.14 *	[1.02–1.27]	0.99	[0.81–1.21]
Temporary (<1 year)	1.18 **	[1.06–1.33]	1.12	[0.92–1.37]	1.41 ***	[1.21–1.65]	1.29	[0.97–1.71]
No contract/other	1.05	[0.97–1.14]	1.01	[0.87–1.18]	1.02	[0.90–1.17]	0.87	[0.67–1.12]
**Sex**								
Women	1.09 ***	[1.04–1.13]	1.09 ***	[1.04–1.13]	1.06	[0.99–1.14]	1.06	[0.99–1.14]
**Age**								
15–29 years	Reference		Reference		Reference		Reference	
30–49 years	1	[0.93–1.04]	1	[0.91–1.04]	1.04	[0.95–1.14]	0.97	[0.87–1.09]
50–65 years	0.93 *	[0.87–1.00]	0.91 *	[0.84–0.98]	1.04	[0.93–1.16]	0.97	[0.85–1.10]
**Job tenure**	1.01	[0.98–1.04]	1.01	[0.98–1.04]	1.09 **	[1.03–1.14]	1.09 ***	[1.04–1.15]
**Job tenure ^2^**	1.00	[0.98–1.01]	1.00	[0.98–1.01]	1.00	[0.97–1.03]	1.00	[0.97–1.03]
**Weekly working hours**	1.05 ***	[1.03–1.07]	1.05 ***	[1.03–1.07]	1.08 ***	[1.05–1.11]	1.08 ***	[1.05–1.12]
**Weekly working hours ^2^**	1.02 ***	[1.01–1.03]	1.02 ***	[1.01–1.03]	1.05 ***	[1.04–1.06]	1.05 ***	[1.04–1.06]
**Income**	0.98	[0.95–1.01]	0.98	[0.95–1.01]	0.93 **	[0.89–0.98]	0.93 **	[0.89–0.98]
**Income ^2^**	1.00	[1.00–1.00]	1.00	[1.00–1.00]	1.00 **	[1.00–1.00]	1.00 **	[1.00–1.00]
**Occupational position**								
Blue collar workers	Reference		Reference		Reference		Reference	
White collar workers	1.04	[0.97–1.10]	1.04	[0.97–1.10]	0.97	[0.88–1.08]	0.97	[0.88–1.08]
Intermediates and lower supervisory	1.05	[0.98–1.12]	1.05	[0.98–1.12]	0.98	[0.88–1.09]	0.98	[0.89–1.09]
Managers and professionals	1.13 ***	[1.07–1.20]	1.13 ***	[1.07–1.20]	1.05	[0.96–1.15]	1.05	[0.96–1.16]
**Company size**								
<10	Reference		Reference		Reference		Reference	
10–249	1.01	[0.97–1.06]	1.01	[0.97–1.06]	0.92 *	[0.85–0.99]	0.92 *	[0.86–0.99]
250+	1.05	[0.99–1.11]	1.05	[0.99–1.11]	0.90	[0.82–1.00]	0.90	[0.82–1.00]
**Number of health events**								
10+	1.30 ***	[1.25–1.35]	1.30 ***	[1.25–1.35]	0.41 ***	[0.38–0.44]	0.41 ***	[0.38–0.44]
**Interaction contract x age**								
Temporary (≥1 year) x 30–49 years			1.00	[0.86–1.16]			1.17	[0.92–1.50]
Temporary (≥1 year) x 50–65 years			1.11	[0.92–1.34]			1.27	[0.94–1.72]
Temporary (<1 year) x 30–49 years			1.06	[0.83–1.36]			1.08	[0.76–1.52]
Temporary (<1 year) x 50–65 years			1.14	[0.81–1.60]			1.31	[0.83–2.07]
No contract/other x 30–49 years			1.02	[0.85–1.22]			1.25	[0.93–1.69]
No contract/other x 50–65 years			1.11	[0.90–1.35]			1.24	[0.88–1.74]
**(Intercept)**	0.42 ***	[0.37–0.49]	0.43 ***	[0.37–0.49]	0.24 ***	[0.19–0.29]	0.25 ***	[0.20–0.31]
**Model information**								
−2loglikelihood	35123.3		35435.5		20577.8		21375.6	
Deviance (%)	−0.88		0.89		−3.73		−3.76	
Likelihood-ratio test	*p* < 0.001		*p* = 0.823		*p* < 0.001		*p* = 0.509	
Individuals	20,240		20,240		20,240		20,240	
Countries	33		33		33		33	

* *p* < 0.05, ** *p* < 0.01, *** *p* < 0.001. ^a^ Model additionally adjusted for working sector. ^2^ Quadratic term.
